# Mental health services for children in care: investigation to elicit outcomes of direct and indirect interventions

**DOI:** 10.1192/bjb.2020.147

**Published:** 2021-10

**Authors:** Samuel Deuchar, Pallab Majumder

**Affiliations:** 1 University of Nottingham, UK

**Keywords:** Looked after children, mental health, evaluation, direct intervention, consultation

## Abstract

**Aims and method:**

The aim of this study was to compare the efficacy of direct therapy and indirect consultation for treating mental health difficulties among looked after children (LAC), and also to identify any demographic or clinical predictor variables for outcomes in this cohort. A retrospective evaluation of mental health outcomes for 104 LAC was conducted. All children received network consultation in combination with or without direct therapeutic work. Outcomes were compared between the groups with and without direct therapeutic intervention.

**Results:**

Those receiving both treatments displayed significantly greater Strengths and Difficulties Questionnaire (SDQ)-rated improvements than those receiving just consultation. Nonetheless, improvements in scores for the latter group were significant relative to baseline. Treatment duration, younger age at referral and start SDQ were all correlated with positive outcomes, while number of address changes predicted higher start SDQ scores.

**Clinical implications:**

Despite the retrospective design of this study, its results can be considered as preliminary findings to guide treatment decisions in LAC.

## Looked after children (LAC) and mental health

Irrespective of circumstances, LAC appear to be disproportionately burdened by an array of adversity, disrupted development and problematic relationships. These challenges commonly manifest as attachment-related disorders, emotional difficulties and maladaptive behaviours. Given the particular vulnerability of this population and the urgency required when dealing with insecure attachment, any research involving an untreated control group would be inherently unethical. Quantitative research in this field has therefore remained sparse, with the vast majority of the literature instead opting to assess case studies and qualitative data. The retrospective nature of the present study means it is able to contribute treatment-related outcome data from a relatively large sample of LAC. Although this naturalistic design is limited in terms of fully controlling confounding variables, it avoids the risk of bias from insufficient blinding and ensures that no child misses out on potentially beneficial treatment.

A comprehensive meta-analysis conducted by Vasileva and Petermann^1^ accumulated data from 22 samples of abused and neglected LAC under 7 years old. Across 11 studies, 38.5% of the 3211 eligible child participants were found to have either motor or cognitive developmental delays; these were consistently more prevalent in children fostered by strangers than in those looked after by kin. Similarly, 39% displayed clinically significant internalising or externalising problems, and only 35% of LAC met criteria for secure attachment. The findings of Tarren-Sweeney^2^ indicate that when mental illness goes untreated during these early years, a child's social and emotional development may be jeopardised, and the possibility of a secure attachment appears to reduce with each placement breakdown. Children that entered care at a later age and were exposed to maltreatment were found to score significantly higher on both the Child Behaviour Checklist and the Assessment Checklist for Children, whereas having a permanent placement was highlighted as an important protective factor. Given the cross-sectional design, however, it was difficult to infer causation, and it is equally possible that children with better mental health are more manageable and thus less at risk of having placements break down.

Attachment security appears to be a crucial factor mediating the mental health outcomes of children placed in foster care. Both internalising problems and frequency of mental illness diagnoses diminished significantly for girls removed from institutional care into foster care, but not for boys, in a sample of Romanian children aged 3 and below.^3^ Subsequent analysis using the strange situation test^4^ revealed that the demonstrated improvements in mental health for fostered girls was fully mediated by the formation of secure attachment. Guyon-Harris et al^5^ examined the longitudinal trajectories of reactive attachment disorder (RAD) based on semi-structured interviews with caregivers. In line with and extending previous conclusions, the sharpest declines in symptoms of RAD were found for children placed in foster care, which was sustained long-term at 8- and 12-year follow-up (73% retention). The vast majority of children with rapidly decreasing symptoms of RAD had been relatively younger when placed in foster care, whereas children entering at an older age displayed persistent and elevated symptoms. Almost all of the original group left in residential care displayed persistent symptoms.

## Direct interventions for mental health difficulties in LAC

The MTFC-P, a direct intervention facilitating healthy, securely attached foster parent–child relationships, included 12 hours of training, daily telephone contact with a consultant and a weekly support group for foster carers, while the child attended weekly therapeutic play group sessions.^6^ Secure and avoidant attachment behaviours were assessed at five time points with 3-month intervals. Pre-schoolers in the MTFC-P condition exhibited significantly more secure and less avoidant behaviours than those in regular foster care. Furthermore, the frequency of secure attachment behaviours increased over time for those receiving the MTFC-P intervention but decreased for the comparison group.

Study on Theraplay have shown statistically significant improvements in communication, interpersonal relationships and behaviour as rated by adoptive mothers, but problem-solving, emotional awareness and general functioning were not found to change.^7^ In a related study, on the other hand, weekly Theraplay sessions delivered to 20 LAC over a period of up to 8 months resulted in no significant change on any of the subscales of the Strengths and Difficulties Questionnaire (SDQ).^8^ Follow-up subgroup analysis indicated that girls showed significantly more teacher-rated improvements than boys in terms of hyperactivity, conduct and prosocial behaviour. Taken together with the findings of McLaughlin and colleagues,^3^ this suggests that girls may perhaps be more able to securely attach to a foster family and so may benefit more substantially from direct intervention.

Dyadic developmental psychotherapy (DDP) is described as a direct, attachment-based intervention involving the delivery of traditional psychotherapy to both the child and their carer.^9^ Wingfield and Gurney-Smith reported that 12 adoptive parents receiving DDP gained increased curiosity, understanding and empathy for their children, while the children reported fewer behavioural problems, improved control over emotions, better relationships with peers and improved sleep.^10^ On the other hand, in spite of progress, a number of parents described the sessions as emotionally exhausting, uncomfortable and upsetting. Feedback from therapists delivering DDP has, however, been very positive. Turner-Halliday and colleagues^11^ reached out to child and adolescent mental health services (CAMHS) in the UK delivering the treatment. DDP was seen as an effective tool for directly tackling complex profiles of mental illness alongside the root causes of both internalising and externalising problems. The inclusion of parents and carers on such an emotionally stimulating journey was seen to indirectly facilitate secure attachment through an improved mutual understanding.

## Multiagency cosultation for LAC mental health

In an attempt to better combat the wide range of complex and overlapping symptoms of behavioural, mental, social and cognitive disorders presented by LAC, a small body of research has explored the utility of multi-agency consultation. Upon recounting a number of particularly challenging cases where successful outcomes only emerged in the wake of a concerted effort from all available support, Sprince^12^ concludes that child therapists have an obligation to appropriately tailor treatments and advice to the specific network of individuals and organisations responsible for the child's care. The consultation model differs from regular therapeutic interventions in that rather than directly treating the child, their network of carers, teachers, social workers and therapists collaborate to tailor solutions to the child's specific needs, difficulties, history and environment.

Swann and York^13^ refer to this multi-agency intervention as ‘THINKSPACE’, succinctly illustrating the creation of a space where clinicians, service providers and stakeholders can come together to share their diverse perspectives, knowledge and skills to conjure up a holistic picture and systemic solution. Consultation sessions dedicate around an hour and a half to each child, beginning with the construction of a narrative outlining their development, diagnoses and family history. Attendees then contribute their unique perspectives, theoretical knowledge and practical experience to generate realistic and appropriate solutions. Swann and York emphasise the importance of using everyday language, having only one key interviewer and avoiding giving the primary carer direct instructions.

Qualitative data for evaluation of the consultation model have been collected from social workers and clinicians by Dimaro, Moghaddam and Kyte.^14^ Feedback from 138 social workers indicated that a vast majority felt that their goals had been suitably addressed by the collaborative consultation sessions, particularly with regard to assessing concerns, understanding behaviours, understanding relationships and planning next steps. However, 37% of those hoping for effective parenting techniques and 41% of those hoping to work more effectively with staff, agencies and local services felt their goal had not been properly addressed. Subsequent focus groups with 12 of the clinicians illuminated a number of key themes. Primarily, they uniformly felt that the sessions allowed them to provide useful, diverse and practical support. They highlighted the utility of clearly defining roles and objectives and spoke positively about the wider systemic effects their input could achieve.

The sole quantitative trial evaluating the effects of consultation on the mental health of LAC was described by Callaghan et al.^15^ Psychiatrists, psychologists and therapists from all three tiers of a UK CAMHS came together to collect collaborative feedback from carers of 45 LAC aged between 4 and 17 years. Outcomes were assessed based on scores on the Health of the Nation Outcome Scales for Children and Adolescents and the SDQ, completed at baseline and 5-month follow-up. Although improvements in total SDQ scores did not quite reach significance, scores on an emotional problems subscale did. This data does rationalise further, more rigorous evaluation of the model but is limited in that it did not include an adequate comparison group, and thus the authors responsible for collecting data were not blinded to experimental procedures. Furthermore, of the 39 carers that completed service satisfaction feedback forms, only 51% felt the intervention offered had been efficacious, and 28% felt their young person had not shown any improvement.

A number of key themes have emerged from the literature exploring mental health presentation and interventions among LAC. Primarily, the formation of secure attachment has consistently been highlighted as a crucial factor mediating mental well-being among this population of particularly vulnerable children. This conclusion emerges from the research contrasting foster care with residential care,^3,5^ the success of treatments targeting child–carer interactions,^7^ and the discrepancy between outcomes for boys and girls,^3,7^ and it is aligned with the apparent predictive power of placement stability and age at entry into care.^2^

Ethical considerations present a major barrier to quantitative research in that withholding potentially efficacious treatment from a vulnerable child can never be justified. However, uncontrolled quantitative data have pointed towards the efficacy of direct work,^6,7^ as has the more abundant qualitative research.^10,11^ The viability of indirect interventions, on the other hand, is far less well evidenced, with the sole quantitative data-set revealing no significant change in SDQ scores^15^ despite promising conclusions drawn from qualitative data.^14^

Therefore, in this study, we aim to test the following key hypotheses.

- Consultation with direct intervention is more efficacious than consultation on its own (H1).
- Consultation on its own is associated with significant SDQ-rated improvements relative to baseline (H2).
- A lower total number of address changes and longer time in current placement (H3), younger age at point of referral (H4) and female gender (H5) predict more positive outcomes.

## Method

### Procedure

In collaboration with a Nottinghamshire-based CAMHS, demographic and treatment-related data for LAC that had accessed the service were collected and analysed (*n* = 437). In pursuit of a suitable outcome measure, only those with both start and end social-worker-rated SDQ scores were included in a subsequent outcome analysis (*n* = 104). Characteristics of the full sample were compared with those of the included sample to determine whether or not it was representative. Permission was then requested and granted to access information regarding presenting problems, diagnoses, address changes and family factors stored on the service's Liquid Logic database for the LAC included in the outcome analysis. Information regarding the nature of treatment for each of these closed cases was also collected, allowing the included sample to be divided into a group receiving just consultation (*n* = 69) and a group receiving both consultation and direct work (*n* = 28).

### Sample

Between May 2002 and June 2019, data were available for a total of 443 referrals at the time of collection. Of these, 437 had sufficient data to be included in a preliminary analysis describing the sample, comprising 247 boys (56.5%) and 189 girls (43.2%), with one missing data point. Out of the full sample of 437 LAC, a total of 104 children (23.8%) had both a start and end SDQ score, thus meeting the inclusion criteria for the treatment outcome analysis. Of the 333 LAC not included, 196 (58.9%) were still undergoing treatment, and the remaining 137 (41.1%) were missing either a start SDQ score, end SDQ score or both. Of the 59 boys (56.7%) and 45 girls (43.3%) included in the follow-up outcome analysis (*n* = 104), 69 received cross-domain consultation on its own (66.3%) and 28 received direct work in addition to their consultation (26.9%); intervention details were not available for the remaining seven LAC (6.7%).

### Measures

The primary outcome measure was scores on the SDQ, a brief and popular tool used for assessing child psychopathology. It is composed of four subscales evaluating difficulties and one accommodating strengths: emotional symptoms, hyperactivity-inattention, conduct problems, peer problems and prosocial behaviours.^16^ Acceptable internal consistency and test–retest stability have been repeatedly demonstrated,^17^ and scores have been found to correlate meaningfully with those of other prominent diagnostic tools.^18^ Change in SDQ score was calculated as the score reported before treatment commenced subtracted by the score following the treatment's conclusion; positive values therefore reflect improvements in SDQ scores.

Regarding categorical predictors, the included LAC were grouped according to ethnicity, gender, disability and adverse experience. Continuous measures included ‘waiting time’, calculated as the number of days between initial referral and the date the child was assigned to a treatment programme, and ‘treatment duration’, operationalised as the number of days between the initial referral and the case closure date. ‘Time in current placement’ was another continuous measure, calculated as the number of days between the most recent address change and the date that data analysis commenced (1 June 2019). ‘Total number of address changes’ included short-term placements and returns to previous addresses, in an attempt to best represent placement stability. Start SDQ score and age at point of referral were two additional and more self-explanatory continuous variables also included as potential predictors.

### Data analysis

Descriptive and frequency statistics were analysed for both the full cross-sectional data-set and the sample included in the outcome analysis. This was followed by a series of one-way analyses of variance (ANOVAs) and independent-samples *t*-tests in order to identify any significant between-group differences. A parallel analysis contrasted the characteristics of the group receiving just consultation with the group receiving both consultation and direct work. This was followed by an independent-samples *t*-test to determine whether the two treatment groups differed from one another in terms of SDQ score changes. A subsequent single-sample *t-*test was used to determine whether SDQ scores within the consultation group improved significantly relative to a baseline of zero. Finally, a predictor analysis considered the full sample of included LAC, commencing with a series of one-way ANOVAs to identify any categorical protective and risk factors. Pearson's R correlation coefficients were then calculated for the relevant continuous measures, with particular attention paid to the factors associated with changes in SDQ score.

### Statement of ethical approval

Ethical approval to conduct this study was not required as the project only involved analysis of existing anonymised data. It was registered in and approved by the Research and Development department of Nottinghamshire Healthcare NHS Foundation Trust.

## Results

### Demographic and clinical findings

In the full sample (*n* = 437), children waited for a mean of 27.7 days (s.d. = 18.4 days, *n* = 317); they had a mean age at point of referral of 11.3 years (s.d. = 4.2 years, *n* = 436) and mean start SDQ score of 17.2 (s.d. = 8.0, *n* = 368). Start SDQ score did not differ significantly between boys and girls (*t* = −0.39, d.f. = 366, *P* = 0.70). A set of one-way ANOVAs contrasted the group characteristics of those included in the follow-up treatment outcome analysis (*n* = 104) with those that had missing SDQ data or were still open cases (Table 1). No significant between-group difference was found for start SDQ score (*F* = 0.512, d.f. = 1, 366, *P* = 0.475), but significant differences were identified for age at referral (*F* = 5.175, d.f. = 1, 434, *P* = 0.023) and waiting time (*F* = 8.366, d.f. = 1, 315, *P* = 0.004). Subsequent independent-samples *t*-tests revealed that with a mean of 12.1 years (s.d. = 4.0) in comparison to 11.0 years (s.d. = 4.2), children with paired outcome data were significantly older at point of referral than those who were excluded (*t* = 2.28, d.f. = 434, *P* = 0.023). With a mean of 21.7 days (s.d. = 11.3) in comparison with 29.1 (s.d. = 19.5), LAC that met the inclusion criteria were also found to have waited significantly less time between referral and choice than excluded participants (*t* = 2.89, d.f. = 315, *P* = 0.004).

**Table 1:** Descriptive statistics contrasting the included with the excluded sample

	Included (*n* = 104)				Excluded (*n* = 333)			
	Mean	s.d.	Range	*n*	Mean	s.d.	Range	*n*
Age at referral (years)	12.1*	4.0	2–17	104	11.0*	4.2	0–17	332
Waiting time (days)	21.7*	11.3	0–48	62	29.1*	19.5	0–104	255
Start SDQ	17.7	8.3	1–36	104	17.0	7.8	0–34	264

*P < 0.05, **P < 0.001.

**Fig. 1:**
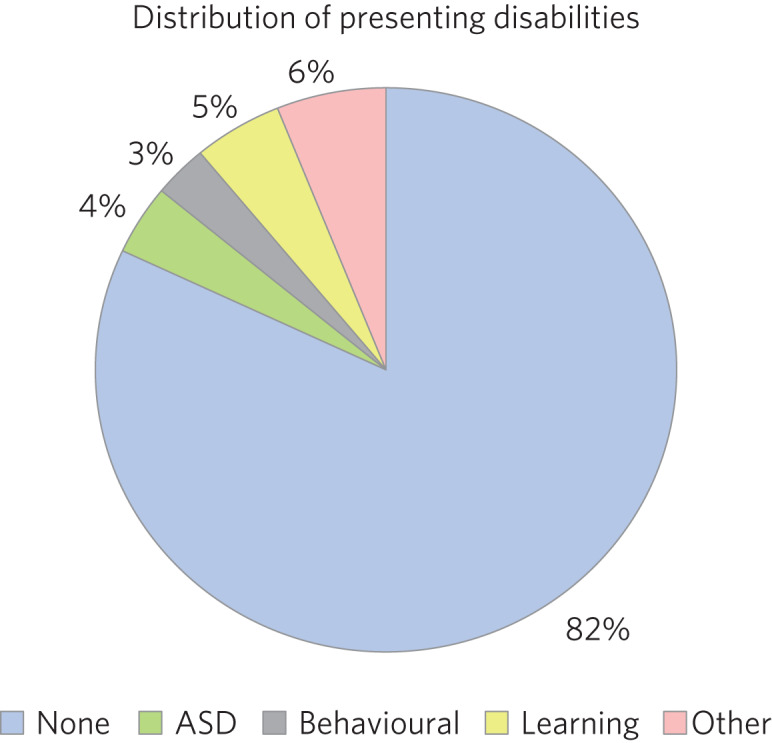
Pie chart graphically illustrating the distribution of presenting disabilities for the included sample of LAC. ASD, autism spectrum disorder.

**Fig. 2:**
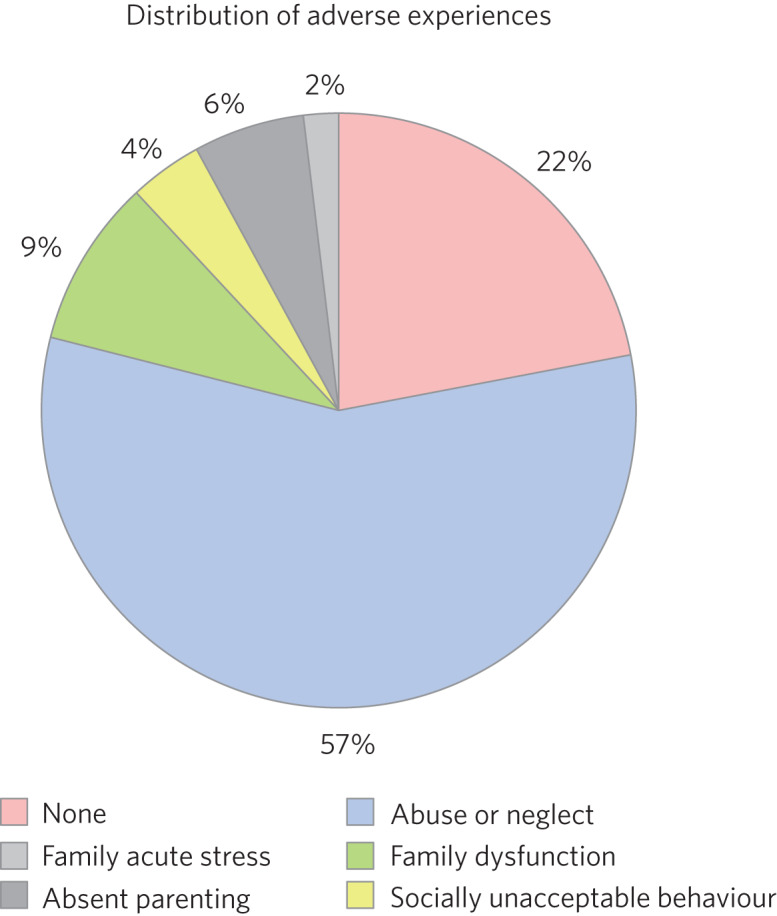
Pie chart graphically illustrating the distribution of adversities experienced by the included sample of LAC at the hands of their birth families.

**Fig. 3:**
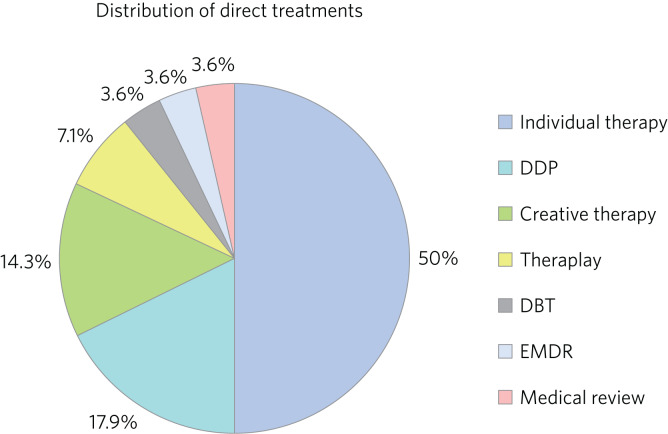
Pie chart graphically illustrating the distribution of direct treatments allocated to the subgroup of included LAC receiving both interventions.

Fifty-nine boys (56.7%) and 45 girls (43.3%) met the inclusion criteria. For the 100 LAC with data available on the Liquid Logic system, presenting disabilities and adverse experiences are displayed graphically in Figs. 1 and 2. The included sample (*n* = 104) presented with a mean start SDQ score of 17.7 (s.d. = 8.3) and a mean end SDQ score of 14.5 (s.d. = 7.4), equating to a mean improvement of 3.1 points (s.d. = 6.6). The mean age at referral was 12.1 years (s.d. = 4.0), the treatment duration was 248.1 days (s.d. = 259.0), time in current placement was 729.7 days (s.d. = 916.8), the total number of address changes was 8.2 (s.d. = 5.9) and, for the 63 LAC with a recorded choice date, the mean waiting time was 21.7 days (s.d. = 11.2) following referral. Sixty-nine of the included participants received cross-domain consultation on its own (66.3%), 28 received direct work in addition to their consultation (26.9%), and intervention details were not available for the remaining seven (6.7%). Fourteen of the participants receiving direct work completed individual therapy (50.0%), five received DDP (17.9%), four were assigned creative therapy (14.3%), two were assigned Theraplay (7.1%) and one was assigned to each of DBT (Dialectical Behaviour Therapy), EMDR (Eye Movement Desensitisation and Reprocessing) and medical review (3.6% each). The distribution of direct treatment interventions is presented graphically in Fig. 3.

### Combined and consultation-only treatment (H1 and H2)

The group of LAC receiving only consultation work (*n* = 69) was composed of 38 males (55.1%) and 31 females (44.9%). They had a mean age of 12.2 years (s.d. = 4.2) at point of referral and a waiting time of 23.3 days (s.d. = 12.2), and their treatment lasted for an average of 177 days (s.d. = 216.2) They had changed address a mean of 7.9 times (s.d. = 6.7) and had been in their current placement for 789.1 days (s.d. = 1032.6). The group receiving both consultation and direct work, on the other hand, was composed of 17 males (60.7%) and 11 females (39.3%). They had a mean age of 11.9 years (s.d. = 3.1), a waiting time of 17.7 days (s.d. = 8.5) and a treatment duration of 401.4 days (s.d. = 305.2). They had changed address a mean of 8.4 times (s.d. = 4.0) and had been in their current placement for 629.9 days (s.d. = 653.1). Those receiving both direct work and consultation were found to have significantly longer treatment duration (*t* = 4.01, d.f. = 91, *P* < 0.001) and a higher start SDQ score (*t* = 2.26, d.f. = 95, *P* = 0.03) than those receiving just consultation (Table 2).

**Table 2:** Descriptive statistics for the two treatment groups

	Consultation (*n* = 69)				Consultation and direct work (*n* = 28)			
	Mean	s.d.	Range	*n*	Mean	s.d.	Range	*n*
Age at referral (years)	12.3	4.2	2–17	69	11.9	3.1	5–16	28
Time in current placement (days)	789.1	1032.6	0–4748	66	629.9	653.1	0–3073	28
Waiting time (days)	23.3	12.2	1–48	43	17.7	8.5	0–34	28
Start SDQ	16.4*	8.4	1–36	69	20.6*	8.2	6–34	15
Treatment duration (days)	177.1**	216.2	0–850	66	401.4**	305.2	0–1205	28
End SDQ	14.3	7.5	2–31	69	15.3	7.7	1–29	27
Change in SDQ	2.1*	6.3	−15 to 18	69	5.4*	7.0	−10 to 18	28

*P < 0.05, **P < 0.001.

**Fig. 4:**
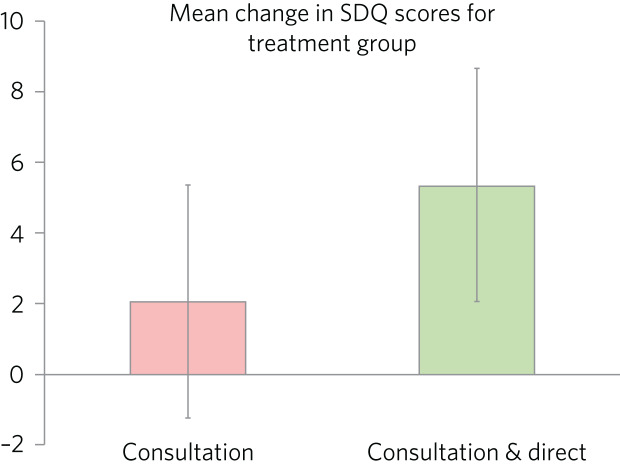
Bar chart graphically illustrating the mean SDQ-rated improvements for both treatment groups, with error bars representing standard deviations.

With a mean change of 5.4 points (s.d. = 7.0) on the SDQ in comparison with 2.1 points (s.d. = 6.3), those receiving both direct and indirect treatment interventions improved significantly more than those receiving just consultation (*t* = 2.26, d.f. = 95, *P* = 0.026). A single-sample *t*-test indicated that those receiving just consultation nonetheless displayed significant SDQ-score improvements (*t* = 2.75, d.f. = 68, *P* = 0.008). These findings are displayed graphically in Fig. 4. A one-way ANOVA found no significant difference in outcomes for the different direct interventions (*F* = 0.65, d.f. = 6, 21, *P* = 0.690). Parallel one-way ANOVAs identified no significant differences when the included sample was grouped by ethnicity (*F* = 0.38, d.f. = 6, 97, *P* = 0.890), disability (*F* = 0.70, d.f. = 4, 95, *P* = 0.593) or adverse experience (*F* = 0.65, d.f. = 5, 94, *P* = 0.662). Across the paired data, mean change in SDQ score did not differ significantly between boys and girls (*t* = 0.41, d.f. = 102, *P* = 0.968).

### Predictor variables of outcome (H3 and H4)

Correlational analysis for the full sample identified a number of factors predicting SDQ-related improvements, as displayed in Table 3. These were: age at point of referral (*R* = −0.22, *P* = 0.027), treatment duration (*R* = 0.20, *P* = 0.049) and start SDQ score (*R* = 0.53, *P* < 0.001). Start SDQ score was also correlated strongly and positively with total number of address changes (*R* = 0.23, *P* = 0.021) and treatment duration (*R* = 0.28, *P* = 0.005). Finally, a negative correlation was found between number of days waiting and total number of address changes (*R* = −0.31, *P* = 0.014).

**Table 3:** Pearson's correlation coefficients for all continuous variables for the included LAC

	Age at Referral	Address changes	Time in current placement	Waiting time	Start SDQ	Treatment duration	Change in SDQ
Age at referral (years)		0.27 (*P* = 0.006)*	−0.23 (*P* = 0.021)*	−0.10 (*P* = 0.417)	−0.09 (*P* = 0.350)	−0.06 (*P* = 0.668)	−0.22 (*P* = 0.027)*
Address changes	0.27 (*P* = 0.006)*		−0.37 (*P* < 0.001)**	−0.31 (*P* = 0.014)*	0.23 (*P* = 0.021)*	−0.12 (*P* = 0.253)	0.05 (*P* = 0.655)
Time in current placement (days)	−0.23 (*P* = 0.021)	−0.37 (*P* < 0.001)**		0.21 (*P* = 0.098)	−0.10 (*P* = 0.345)	0.12 (*P* = 0.256)	0.023 (*P* = 0.822)
Waiting time (days)	−0.10 (*P* = 0.417)	−0.31 (*P* = 0.014)*	0.21 (*P* = 0.098)		−0.02 (*P* = 0.885)	−0.06 (*P* = 0.668)	0.08 (*P* = 0.512)
Start SDQ	−0.09 (*P* = 0.350)	0.23 (*P* = 0.021)*	−0.10 (*P* = 0.345)	−0.02 (*P* = 0.885)		0.28 (*P* = 0.005)*	0.53 (*P* < 0.001)**
Treatment duration (days)	−0.06 (*P* = 0.668)	−0.12 (*P* = 0.253)	0.12 (*P* = 0.256)	−0.06 (*P* = 0.668)	0.28 (*P* = 0.005)*		0.20 (*P* = 0.049)*
Change in SDQ	−0.22 (*P* = 0.027)*	0.05 (*P* = 0.655)	0.023 (*P* = 0.822)	0.08 (*P* = 0.512)	0.53 (*P* < 0.001)**	0.20 (*P* = 0.049)*	

*P < 0.05, **P < 0.001.

## Discussion

In summary, the LAC with paired outcome data (*n* = 104) differed significantly from the rest of the sample (*n* = 333) in that they were older at the point of referral (12.1 years *v.* 11.0 years) and waited for less time between referral and appointment to a treatment programme (27.1 days *v.* 29.1 days). It is unclear why children referred to the CAMHS at an older age were more likely to have completed treatment and received both start and end SDQ scores. For the latter discrepancy, on the other hand, it is plausible that shorter waiting times may result in faster recovery, meaning treatment is more likely to be concluded for those that received it quickly.

The children allocated both consultation and direct work (*n* = 28) differed significantly from those receiving just consultation (*n* = 69) in that their treatment lasted longer and they commenced with a higher start SDQ score (20.6 *v.* 16.4). Given that the former group were receiving two forms of treatment as opposed to one, it is understandable that the overall duration would be longer. As for the higher start SDQ score, this discrepancy is likely to reflect the unrandomised group allocation. Treatments were instead allocated based on the needs of the LAC; those with more severe presentations and exposed to more adversity were more likely to receive both forms of treatment. One important strength of the present analysis is that outcomes were assessed and scored by the child's social worker, a third party with no conceivable bias towards observing an improvement.

Despite more severe presentations, the group receiving both treatments displayed significantly greater improvements in SDQ scores than the group receiving just indirect work (5.4 *v.* 2.1), thus confirming the primary hypothesis (H1). As outcomes for the various direct interventions did not differ significantly from one another, this finding can be interpreted as qualitative support for all the included direct treatment programmes. In particular, it extends the findings of Weir et al^7^ in their endorsement of the efficacy of Theraplay, but it contradicts Francis, Bennion and Humrich,^8^ who did not find a significant change in SDQ score. It also provides concrete quantitative data to validate the qualitative findings of DDP research.^10,11^ Furthermore, it forms a preliminary benchmark for future research evaluating the utility of both individual and creative therapy, which is yet to be evidenced in a population of LAC. Conclusions regarding the other three direct interventions are more tentative given that only one child received each of DBT, EMDR and medical review, but the outcomes are nonetheless promising.

Consultation was found to be independently efficacious at moderating SDQ scores relative to a baseline of zero, thus confirming the secondary hypothesis (H2). This finding opposes that of Callaghan and colleagues,^15^ who did not find any significant change in SDQ score following consultation, perhaps owing to their smaller sample size or the shorter treatment duration. On the other hand, it aligns more readily with the qualitative data reported by Dimaro, Moghaddam and Kyte.^14^ This finding for the consultation-only group, however, should be viewed with some caution. Clinicians appear to have made a judgement that these children did not merit a direct intervention, most likely owing to less severe presentation. Consequently, the finding that CAMHS consultation was of benefit is promising. However, the other finding that this group showed less significant change than the group receiving both treatments may imply that these children required additional therapeutic intervention, which was not necessarily within the specialist CAMHS remit. The findings, therefore, may rekindle the debate on the needs and resource implications of therapeutic services, often offered by other agencies such as social care and the third sector, that have been substantially lost in recent years in the UK.

The ingredients of the consultation model used in the study sample consisted of collaboration and cooperation among a specific network of carers, professionals and organisations to create a thinking space where the complex needs of the LAC could be assessed, understood and managed by mutual support, clarification of roles, common understanding and practical steps. This is compatible with previous work that endeavoured to illustrate the consultation model.^12,13^ Unfortunately, there are no data available on the rationale or indication for offering indirect intervention. Future service-based clinical studies may consider looking into the rationale for choosing consultation as a treatment, for example, data on goal setting and management plans following initial evaluation. Quantifying gradual systemic improvement in general functioning and long-term life trajectories of LAC as a result of indirect intervention remains a challenge to achieve through retrospective or short-term quantitative research.

Contrary to expectations, hypothesis three (H3) was not supported by the present data-set in that greater length of time in current placement did not predict more substantial SDQ-score improvements, nor was number of address changes found to moderate these improvements. However, in line with the findings of Tarren-Sweeney,^2^ the present analysis did reveal start SDQ score to be positively correlated with number of address changes. The causal directionality of this well-evidenced correlation between mental health presentation severity and frequency of placement breakdown is still unclear.

Hypothesis four (H4) was supported by the present analysis, with age at point of referral negatively correlated with change in SDQ score. This demonstrates the benefits of intervention at a younger age and somewhat aligns with the findings of Tarren-Sweeney^2^ and Guyon-Harris et al,^5^ both of whom endorse younger age at entry into care as an important protective factor. Probably also driven by an increased propensity to develop secure attachment,^3,5^ it is apparent that parallel to younger age at entry into care, early intervention from CAMHS similarly predicts more substantial improvements. In contrast to previous literature endorsing a gender discrepancy,^3,8^ the present data-set displayed no significant difference in start SDQ score across the full sample, nor did boys and girls included in the outcome analysis differ significantly. This lack of support for hypothesis five (H5) speaks positively towards the efficacy and reliability of both consultation and direct intervention. Similarly, treatment outcomes were equivalent across ethnicity, disability and adverse experience, further endorsing the cross-contextual efficacy of both consultation and direct work.

### Limitations

Although the present data-set does provide convincing support for both treatment programmes, conclusions cannot be made regarding their relative efficacy as both groups received consultation. Future experimental research should deliver indirect interventions and direct interventions on their own, to two separate groups. Where in the past the risk of one or both treatments being ineffective may have deemed an investigation of this kind unethical, it can be rationalised by the improvements displayed by both groups in the present study. It is possible for the inclusion criteria to be biased towards including individuals that have responded positively to treatment, who are more likely to have had their case closed and to have completed an end SDQ. However, a noteworthy proportion of the included sample appear to have concluded treatment on turning 18 years of age. This would also explain why the included sample had a mean older age. Although previous research does support the inference that treatment-related improvements are attributable to the facilitation of secure attachment, attachment security was not included as an outcome measure in this study. Given widespread evidence for attachment as a crucial mediating factor,^5,6^ it would be advisable for future research to include attachment security as an outcome measure. This may include considering incorporating the strange situation test^4^ to characterise the attachment between a child and their carer as either secure, avoidant or resistant.

### Implications

The literature review that commenced this report isolated the formation of secure attachment as a crucial mediating factor in determining the mental well-being of LAC. It also outlined support for direct work in the promotion of mental health in this population and identified a gap in the LAC literature, with indirect consultation yet to be suitably and quantitatively assessed. This report is the first of its kind to contrast direct and indirect treatment-related outcomes for LAC, and it endorses the efficacy of both. Younger age at entry into care and early treatment are further solidified as key protective factors for mental health of LAC, whereas the previously reported effect of gender is undermined. Despite a number of limitations, these findings provide an important quantitative benchmark to guide treatment decisions and future research exploring the efficacy of interventions for this particularly vulnerable population of children.

## Data Availability

The data that support the findings of this study are available from the corresponding author, P.M., upon reasonable request.

## About the authors

**Samuel Deuchar**, MSc, is a postgraduate researcher at the School of Psychology, University of Nottingham, UK; **Pallab Majumder**, MBBS, MRCPsych, MD, PhD, is an Honorary (Consultant) Assistant Professor at the University of Nottingham, and Consultant Child and Adolescent Psychiatrist at Nottinghamshire Healthcare NHS Foundation Trust, UK.
